# Comparison of the drug retention and reasons for discontinuation of tumor necrosis factor inhibitors and interleukin-6 inhibitors in Japanese patients with elderly-onset rheumatoid arthritis—the ANSWER cohort study

**DOI:** 10.1186/s13075-021-02496-w

**Published:** 2021-04-15

**Authors:** Sadao Jinno, Akira Onishi, Maureen Dubreuil, Motomu Hashimoto, Wataru Yamamoto, Koichi Murata, Tohru Takeuchi, Takuya Kotani, Yuichi Maeda, Kosuke Ebina, Yonsu Son, Hideki Amuro, Ryota Hara, Masaki Katayama, Jun Saegusa

**Affiliations:** 1 Department of Rheumatology and Clinical Immunology, Kobe University Graduate School of Medicine, 7-5-2 Kusunoki-chou Kobe-shi, Hyogo, 650-0017 Japan; 2 Section of Rheumatology, Department of Medicine, Boston University School of Medicine, Boston, MA USA; 3 VA Boston Healthcare System, Boston, MA USA; 4 Department of Advanced Medicine for Rheumatic Diseases, Kyoto University Graduate School of Medicine, Kyoto, Japan; 5 Department of Internal Medicine IV, Osaka Medical College, Osaka, Japan; 6 Department of Respiratory Medicine and Clinical Immunology, Osaka University Graduate School of Medicine, Osaka, Japan; 7 Department of Musculoskeletal Regenerative Medicine, Osaka University, Graduate School of Medicine, Osaka, Japan; 8 First Department of Internal Medicine, Kansai Medical University, Osaka, Japan; 9 The Center for Rheumatic Diseases, Department of Orthopaedic Surgery, Nara Medical University, Nara, Japan; 10 Department of Rheumatology, Osaka Red Cross Hospital, Osaka, Japan

**Keywords:** ANSWER cohort, Biological disease-modifying antirheumatic drugs, Drug retention, Elderly onset rheumatoid arthritis

## Abstract

**Background:**

This multi-center, retrospective study aimed to clarify retention rates and reasons for discontinuation of either tumor necrosis factor inhibitors (TNFi) or interleukin-6 inhibitors (IL-6i) in patients with elderly-onset rheumatoid arthritis (EORA).

**Methods:**

Patients with rheumatoid arthritis (RA) enrolled in a Japanese multicenter observational registry between 2011 and 2020 were included. EORA was defined as RA with onset at 60 or over. To adjust confounding by indication for treatment with TNFi or IL-6i, a propensity score based on multiple baseline characteristics variables was used to compare the drug retention and causes for discontinuation between TNFi and IL-6i. Adjusted cumulative incidence of drug discontinuation for each reason was compared between the two groups using the Fine-Gray model.

**Results:**

Among a total of 9,550 patients in the registry, 674 TNFi and 297 IL-6i initiators with EORA were identified. Age, the proportion of females, disease duration, and baseline disease activity at the time of TNFi or IL-6i initiation were similar between the two groups. After adjusting for differences in baseline characteristics between the two groups, overall drug discontinuation was significantly lower in the IL-6i as compared to the TNFi (HR = 0.71, 95%CI = 0.59–0.86, *p* < 0.001). The adjusted cumulative incidence of discontinuation due to lack of effectiveness was lower with the IL-6i (HR = 0.46, 95%CI = 0.33–0.63, *p* < 0.001) while those due to adverse events (HR = 0.82, 95%CI = 0.56–1.18, *p* = 0.28) or achievement of clinical remission (HR = 1.09, 95%CI = 0.62–1.91, *p* = 0.76) were similar between the two groups.

**Conclusions:**

In EORA patients initiating a TNFi or IL-6i, significantly higher drug retention was observed with IL-6i. Discontinuation due to lack of effectiveness was significantly less frequent in IL-6i while discontinuations due to adverse event or achievement of clinical remission were similar between the two groups.

## Introduction

Rheumatoid arthritis (RA) among elderly people is an increasingly important health concern. A recent large RA registry in the USA showed that approximately one-fourth of the enrolled patients were diagnosed after the age of 60 years [1]. In a Swiss prospective observational cohort for early RA and undifferentiated arthritis (disease duration after the first symptom < 1 year), the peak age at disease onset was between 50 and 60 years and was ≥60 years in 38.2% of the 592 patients [2]. In Japan, where the population has aged rapidly in recent decades, one of the large registry studies showed the peak age at onset of RA has shifted from 50–59 to 60–69 years of age over the past decade [3]. In fact, it is not uncommon to see patients who develop RA over the age of 70 or even 80 in a typical Japanese rheumatology practice.

Elderly onset-rheumatoid arthritis (EORA) is usually defined as disease onset after 60 years of age [4]. Patients with elderly-onset rheumatoid arthritis (EORA) can present with higher disease activity at diagnosis, greater disability, and a greater burden of comorbidities than those with young-onset rheumatoid arthritis (YORA) [4–7]. It is not entirely clear why these discrepancies arise between the two age groups, but one study reported EORA patients had higher IL-6 levels and lower TNF- alpha levels as compared to YORA patients [8]. Furthermore, previous studies showed the distribution of the human leukocyte antigen-DRB1 genotypes were different between EORA and YORA [9, 10]. These findings suggest different cytokines may be involved in pathogenesis between the two age groups.

With a potentially growing number of EORA patients who may have a different cytokine profile from YORA patients, it is imperative to know which biologic disease modifying anti-rheumatic drugs (bDMARDs) can be used more effectively and safely among them. However, there have been no randomized controlled trials comparing the efficacy and safety of different bDMARDs among EORA patients. Randomized controlled trials primarily recruit healthy or single-disease volunteers rather than elderly patients or those with comorbidities [11]. Thus, cohort-based observational studies may be more suitable to investigate the performance of bDMARDs among unique population such as EORA. There have been a few observational studies comparing the effectiveness of bDMARDs among elderly patients with RA [12, 13]. These studies included not only EORA but young-onset RA who had relatively long disease duration.

Given that drug retention can be a proxy to both safety and effectiveness of bDMARDs [14–16], we hypothesized comparison of drug retention is a reasonable way to assess the efficacy and safety of bDMARDs among EORA patients. The objective of the study was to investigate drug retention and reasons for discontinuation of TNFi and IL-6i among EORA by utilizing the data from multicenter observational cohort in Japan.

## Methods

### Study design and data source

The Kansai Consortium for Well-being of Rheumatic Disease Patients (ANSWER) cohort is an observational multi-center registry of patients with RA in the Kansai district of Japan. Data from patients at seven institutes (Kyoto University, Osaka University, Osaka Medical College, Kansai Medical University, Kobe University, Nara Medial University, and Osaka Red Cross Hospital) were included. ANSWER is an ongoing prospective cohort study of adult patients with RA [13, 17]. Serial disease assessments including laboratories and treatment history were recorded. The data from 2011 to 2020 were retrospectively analyzed. In this study, we included all patients whose RA onset was at age 60 or over. Patients with RA fulfilled the 1987 American College of Rheumatology (ACR) or 2010 ACR/European League Against Rheumatism (EULAR) criteria. Patients were treated according to the Japan College of Rheumatology guideline [18], similar to the EULAR and ACR guidelines, first with a conventional synthetic DMARD (csDMARD), primarily methotrexate with or without glucocorticoids, followed by the addition of bDMARDs or other csDMARDs, using the treat-to-target approach [19, 20]. This study used data on the following bDMARDs: TNFi (adalimumab, certolizumab pegol, etanercept, golimumab, infliximab) and IL-6i (tocilizumab or sarilumab). Baseline demographic data including age, sex, tender joint count, swollen joint count, patient global assessment (PtGA), physician global assessment (PGA), baseline disease activity (disease activity score in 28 joints-erythrocyte sedimentation rate [DAS28-ESR], clinical disease activity index [CDAI], and simple disease activity index [SDAI]), disease duration of RA, current bDMARD use, number of previously administered bDMARDs, reasons for discontinuation of bDMARDs, dates of both starting and discontinuing bDMARDs, concomitant use of methotrexate, glucocorticoids, sulfasalazine, and other csDMARDs such as leflunomide, bucillamine, iguratimod, and tacrolimus were collected. Other baseline demographic features such as baseline C-reactive protein (CRP), rheumatoid factor (RF), and anti-cyclic citrullinated peptide antibody (ACPA) positivity, and Health Assessment Questionnaire disability index (HAQ-DI) score were also collected. Baseline demographic data was collected within 90 days prior to the date each bDMARD was initiated while baseline disease activity was measured at the time of bDMARD initiation. We have included all treatment with bDMARDs in each patient and reflected in the number of previously administrated bDMARDs.

### Outcome of interest

The primary outcome of interest, drug retention after initiation of TNFi or IL-6i, was evaluated using the time until definitive treatment discontinuation. Temporary discontinuations followed by reintroduction of the same medication were not recorded as discontinuations. As a part of requirement for cohort participation, physicians were mandated to report a reason for discontinuation of bDMARDs. The reasons for discontinuation were as follows: drug inefficacy, achievement of clinical remission, toxic adverse events, patient preference (including financial reasons), loss to follow-up, and other. The secondary outcomes included adjusted cumulative incidence of specific reasons for drug discontinuation: lack of effectiveness, adverse events, or RA remission after initiation of a TNFi or IL-6i.

The study was approved by the ethics committee of Kobe University (approval number 1738) as well as the ethics committees of all participating institutions. The study was conducted in accordance with the Declaration of Helsinki. In our institute, the institutional review board waived the requirement for patients’ informed consent because this study utilized only existing data collected in clinical practice. The opportunity to refuse participation in the research was ensured for the research subjects. The study was approved by the institutional review board of all 7 institutes.

### Statistical analysis

A propensity score approach was used to account for differences in potential confounding factors. A propensity score was recalculated at the time of initiating each bDMARD. A logistic regression model was used to calculate the propensity score defined as the probability of initiation of TNFi or IL-6i based on patient covariates. Prespecified potential confounding factors and predictors of the outcome were age, sex, RA duration, baseline CDAI, RF or ACPA positivity, concomitant glucocorticoid, methotrexate, sulfasalazine and other csDMARDs, and number of previously administered bDMARDs [21–24]. The analyses were based on inverse probability of treatment weighting to reduce the variability of weights and standard errors of estimated treatment effects. We used the Cox proportional model for the primary outcome, bDMARD retention, and the Fine-Gray hazard competing risk regression model for adverse events, lack of effectiveness, and remission and accounted for clustering effects by individual [25]. To account for missing data, we used multiple imputations by a chained equation, using all other variables to impute any missing values for variables included in the logistic regression model. We generated 5 independent imputed datasets. For each dataset, we estimated propensity score from the logistic model and pooled the resulting parameters according to Rubin’s rules [26]. Lastly, we performed the Cox proportional model for bDMARD retention among those who did not respond to initial TNFi as a subgroup analysis. Statistical analyses were performed using SAS version 9.3 and STATA version 13.1 (StataCorp, Texas, USA). *P* < 0.05 were considered statistically significant.

## Results

### Patient characteristics

The study population was selected from all patients with RA in the ANSWER cohort (*n* = 9,550) who fulfilled the inclusion criteria during the study period. A total of 674 TNFi and 297 IL-6i initiators with EORA were identified. Baseline demographics, disease characteristics, and concomitant therapies were mostly similar between the groups (Table 1). The median age of EORA at the time of TNFi or IL-6i initiation was similar. The proportion of females was similar between the two groups. The median disease duration was slightly longer in the IL-6i initiators. The median CRP was significantly higher in the IL-6i initiators. The percentage of RF and ACPA positivity was similar between the two groups. Baseline DAS28-ESR, SDAI, and CDAI, compared to those with lowand HAQ-DI score were similar between the two groups. Methotrexate usage was less frequent in the IL-6i initiators. Glucocorticoid usage was more frequent in the IL-6i. Sulfasalazine usage was similar while other csDMARDs usage was more frequent in the IL-6i. The TNFi group received bDMARDs as the first agent more frequently as compared to the IL-6i. Of the TNFi-treated patients, 177 (26.3%) initiated etanercept, 114 (16.9%) adalimumab, 83 (12.3%) certolizumab pegol, 62 (9.2%) infliximab, and 238 (35.3%) golimumab. Of the IL-6i-treated patients, 272 (91.6%) initiated tocilizumab and 25 (8.4%) sarilumab.

**Table 1:** Clinical characteristics of elderly-onset RA at initiation of TNFi or IL-6i

Characteristic	TNFi (*n* = 674)	IL-6i (*n* = 297)	*P* value
Age, median years (IQR)	71 (67–77)	72 (67–77)	0.34
Female sex, *n* (%)	513 (76.1)	221 (74.4)	0.57
Disease duration, median months (IQR)	32 (12–77)	39 (16–79)	0.12
CRP (mg/dL), median (IQR)	1.0 (0.20–3.1)	1.74 (0.30–4.5)	0.02
RF-positive, *n* (%)	452 (72.6)	200 (73.5)	0.81
ACPA-positive, *n* (%)	396 (73.1)	172 (74.8)	0.66
Tender joint count, median (IQR)	3 (1–6)	2 (1–5)	0.47
Swollen joint count, median (IQR)	3 (1–6)	2 (1–4)	0.36
PtGA VAS (0–100 mm), median (IQR)	54 (30–74)	54 (30–74)	0.82
PGA VAS (0–100 mm), median (IQR)	34 (19–55)	35 (19–54)	0.99
DAS28-ESR, median (IQR)	4.6 (3.7–5.5)	4.6 (3.6–5.5)	0.66
SDAI, median (IQR)	17 (11–25)	16 (10–25)	0.75
CDAI, median (IQR)	16 (10–23)	14 (10–22)	0.29
HAQ-DI, median (IQR)	1.0 (0.38–1.75)	1.0 (0.38–1.88)	0.65
Concurrent methotrexate use, *n* (%)	417 (61.9)	140 (47.1)	< 0.001
Methotrexate dosage (mg/week), median (IQR)	8 (6–10)	8 (6–10)	0.99
Glucocorticoid use, *n* (%)	259 (38.4)	141 (47.4)	0.009
Glucocorticoid dosage (mg/day), median (IQR)	5.0 (3.0–7.0)	5.0 (4.0–8.0)	0.52
Sulfasalazine use, *n* (%)	152 (22.6)	70 (23.6)	0.74
Other csDMARDs use, *n* (%)	110 (16.4)	73 (24.8)	0.003
1st bDMARD (%)	430 (63.8)	127 (42.8)	< 0.001
2nd bDMARD (%)	164 (24.3)	88 (29.6)	0.12
≥3rd bDMARD (%)	80 (11.9)	82 (27.6)	< 0.001

Data are no. (%) patients or median IQR

Demographic and clinical characteristics at initiation of TNFi or IL-6i summarized as median for continuous data and as numbers (percentages) for categorical data. *T* test or Wilcoxon signed-rank test and the chi-squared test were used to compare the clinical characteristics between the 2 groups for continuous variables and categorical variables, respectively. *ACPA* anti-citrullinated protein antibodies, *bDMARDs* biological disease-modifying antirheumatic drugs, *csDMARDs* conventional synthetic DMARDs, *CDAI* clinical disease activity index, *CRP* C-reactive protein, *DAS28-ESR* Disease Activity Score 28-erythrocyte sedimentation rate, *EORA* elderly-onset rheumatoid arthritis, *IL-6i* interleukin-6 inhibitors, *HAQ-DI* Health Assessment Questionnaire Disability Index, *PtGA* patient global assessment, *PGA* physician global assessment, *RF* rheumatoid factor, *SDAI* simplified disease activity index, *TNFi* tumor necrosis factor inhibitors, *YORA* young-onset rheumatoid arthritis, *VAS* visual analogue scale

### Drug retention and specific causes for discontinuation

**Fig. 1:**
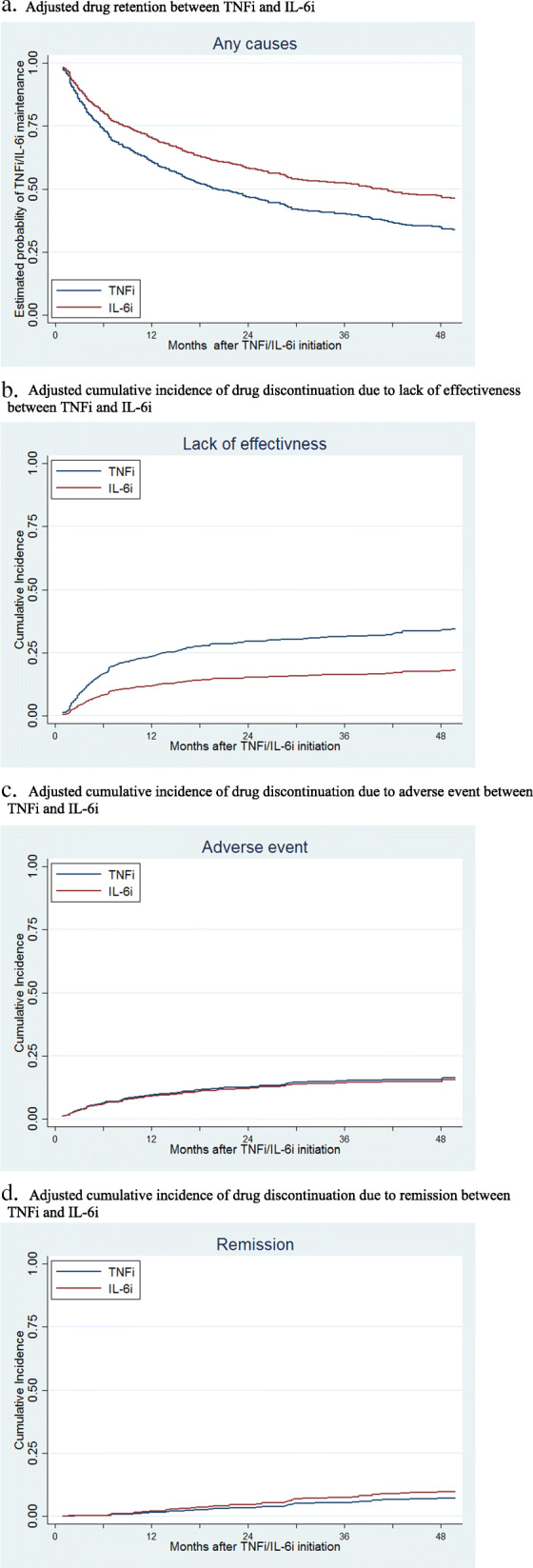
Adjusted drug retention and adverse event among TNFi and IL-6i patients TNFi tumor necrosis factor inhibitors, IL-6i interleukin-6 inhibitors. **a** Adjusted drug retention between TNFi and IL-6i. **b** Adjusted cumulative incidence of drug discontinuation due to lack of effectiveness between TNFi and IL-6i. **c** Adjusted cumulative incidence of drug discontinuation due to adverse event between TNFi and IL-6i. **d** Adjusted cumulative incidence of drug discontinuation due to remission between TNFi and IL-6i

The median follow-up duration was 418 days in the TNFi and 497 days in the IL-6i. After adjustment for other confounders, IL-6i use was associated with significantly lower overall drug discontinuation as compared to the TNFi (HR = 0.71, 95%CI = 0.59–0.86, *p* < 0.001) (Fig. 1a). Adjusted cumulative incidence of drug discontinuation for each specific cause was compared between the two groups. The incidence of drug discontinuation due to lack of effectiveness was significantly lower in the IL-6i group (HR = 0.46, 95%CI = 0.33–0.63, *p* < 0.001) (Fig. 1b). The incidence of drug discontinuation due to adverse event (HR = 0.82, 95%CI = 0.56–1.18, *p* = 0.28) and clinical remission (HR = 1.09, 95%CI = 0.62–1.91, *p* = 0.76) were similar between the two groups after adjustment for the confounders (Fig. 1c and d). Furthermore, we performed a subgroup analysis among EORA patients who did not respond to initial TNFi (*n* = 197). IL-6i (*n* = 106) use was associated with significantly lower overall drug discontinuation as compared to TNFi (*n* = 91) (HR = 0.62, 95%CI = 0.41–0.92, *p* = 0.02).

## Discussion

In this analysis of a large Japanese RA registry, overall drug discontinuation and discontinuation specifically due to lack of effectiveness were significantly lower in the IL-6i as compared to the TNFi. Discontinuations due to adverse event or achievement of clinical remission were similar between the two groups. To the best of our knowledge, this is the first report in the real-world setting comparing the drug retention and reasons for discontinuation between TNFi and IL-6i among persons with EORA.

Patients who initiated IL-6i in the present study had longer drug retention than those who initiated TNFi, which may be related to differences observed in clinical effectiveness. In fact, drug discontinuation due to lack of clinical effectiveness was significantly lower in the IL-6i group. The reasons for the difference are considered as follows. First, EORA patients could have inflammation mainly driven by high IL-6 levels, indicating blocking IL-6 is a reasonable way of reducing an inflammatory cascade among EORA patients. In the present study, the median baseline CRP was significantly higher in the IL-6i as compared to the TNFi (1.74 mg/dL versus 1.02 mg/dL; *p* = 0.02). Several studies have found RA patients with high IL-6 levels had a greater response to IL-6i compared to TNFi [27–30]. Most recently, a post hoc analysis by Boyapati et al. evaluated whether baseline IL-6 levels are predictive of sarilumab treatment responses in 2 phase III studies [29]. Patients with high baseline IL-6 levels (all ≥3 times the upper limit of normal; *n* = 100) had higher disease activity at baseline than those with low IL-6 levels (*n* = 100). The magnitude of clinical improvement over 24 weeks with sarilumab versus adalimumab was greater in patients with high baseline IL-6 levels compared to those with low baseline IL-6 levels. Second, EORA patients often have large joint involvement associated with higher serological inflammatory markers and more functional disabilities [4–7]. In such cases, agents that effectively control large joints inflammation would be a reasonable option. A group from Japan compared the effectiveness of IL-6i with TNFi in the treatment of RA patients who have knee joint involvement [31]. The patients who had knee joint involvement and were treated with an IL-6i had greater improvement of CDAI from baseline (*n* = 95, ΔCDAI 15.0 ± 10.8; mean ± SD) compared to those treated with TNFi (*n* = 148, ΔCDAI 11.4 ± 10.3; mean ± SD) at 12 weeks (*P* = 0.003). These findings suggest IL-6i may be more effective in EORA patients, especially with large joint involvement. Additionally, EORA patients may have concurrent renal insufficiency that could limit the use of methotrexate co-therapy. EORA patients also may not tolerate methotrexate due to comorbidities or adverse events. In a Canadian population-based study, increasing age was associated with an increased tendency towards methotrexate discontinuation in newly diagnosed RA patients [32]. Hence, bDMARD monotherapy may be necessary in EORA patients. In such cases, IL-6i may be a better approach than TNFi based on its efficacy without concurrent methotrexate therapy [33].

Previous studies that compared the efficacy of TNFi with IL-6i were primarily based on patients with YORA. The randomized controlled phase IV ADACTA trial in patients with RA (mean age 53) who were intolerant of methotrexate or for whom continued therapy with methotrexate was inappropriate demonstrated superiority of tocilizumab monotherapy over adalimumab monotherapy for change in the DAS28-ESR from baseline to week 24 [33]. More tocilizumab-treated than adalimumab-treated patients achieved remission according to the DAS28-ESR (DAS28 < 2.6) and the CDAI (CDAI ≤ 2.8). In real-world settings, a recent study involving 11,505 patients (median age 54) from 7 European registries demonstrated that CDAI low disease activity and remission were similar between TCZ with or without csDMARDs and TNFi with csDMARDs [34]. On the other hand, several observational studies have found TCZ was associated with longer drug retention and/or increased effectiveness than TNFi [35–37]. This discrepancy may be due to the heterogeneity across studies in terms of study design, outcomes, and methodology.

A subgroup analysis showed IL-6i was associated with significantly higher drug retention as compared to a TNFi among EORA patients who did not respond to initial TNFi, suggesting agents with another mode of action such as IL-6i may be more effective than a second TNF among initial TNFi non-responders. Our results are in support of other reports on YORA patients that demonstrated better efficacy using an agent with another mode of action rather than a second TNFi [38]. These results suggest treatment for initial TNFi non-responders among EORA patients should be agents with another mode of action rather than a second TNFi.

Adverse event discontinuation was similar between the TNFi and IL-6i in our population. Our results are in support of a previous observational study that reported a similar safety profile between the two medication classes [39–41]. A propensity score-matched study using large claims data from US Medicare found that the risk of severe infections was not different between tocilizumab and TNFi initiators (combined HR 1.05, 95% CI 0.95 to 1.16) [39]. Other studies evaluating the safety profile between the two agents were mostly based on RA patients younger than 60 year of age. A prospective cohort study using a Japanese RA registry showed no significant difference in the severe infection rate with tocilizumab versus TNFi use (HR 2.23, 95% CI 0.93 to 5.37) [40]. On the other hand, an observational cohort study using data from the British Society for Rheumatology Biologics Register for Rheumatoid Arthritis showed an increased risk (HR 1.22, 95% CI 1.02 to 1.47) in tocilizumab compared with etanercept for severe infection, defined as an infection resulting in death, hospitalization or requiring intravenous antimicrobial therapy [42]. Such discrepancies could arise from differences in study population, comparison group, and outcome definition. Our study, along with another study that investigated the safety of IL-6i among elderly RA population [39], provides additional data to the literature that IL-6i therapy is generally well tolerated among them. Patient-specific risk factors such as comorbidities may be more important on the risk of severe infection than the choice of bDMARDs between TNFi and IL-6i [41].

The present study had some limitations. First, comorbidities such as diabetes mellitus or respiratory diseases were not adjusted as confounders between the two groups since the registry does not include comorbidities information. Healthier patients could be selected more frequently for IL-6i treatment as compared to TNFi (or vice versa). However, we have adjusted for available confounders such as disease duration, baseline disease activity, or concomitant use of glucocorticoids between the two groups. Second, the judgment and reasons for discontinuation (such as lack of effectiveness or remission) depended on the decisions of each physician without standardized criteria. Third, the difference between intravenous and subcutaneous bDMARDs could not be determined. Fourth, the study population was predominantly Japanese. Our results may not be generalizable to other patient populations due to differences in patient factors or practice patterns. Fifth, number of previously administrated bDMARDs. Lastly, drug retention was used for outcome assessments in the present study while response criteria remain standard outcome measures for clinical trials.

The strengths of this study include the use of data from a large multi-center cohort of EORA patients with prospectively collected detailed longitudinal clinical data including both clinical outcomes and adverse events. We used sophisticated statistical models adjusting for potential confounders using inverse probability of treatment weighting and the Fine-Gray hazard competing risk regression model.

In conclusion, in EORA patients initiating a TNFi or IL-6i, significantly higher drug retention was observed with IL-6i. Discontinuation due to lack of effectiveness was significantly lower with IL-6i while discontinuations due to an adverse event or achievement of clinical remission were similar between the two groups. Further investigation is warranted in other datasets to draw more conclusive estimates on the comparative effectiveness and safety of bDMARDs in patients with EORA.

## Data Availability

The datasets generated and/or analyzed during the current study are not publicly available. Patients did not provide consent for raw data sharing during the data collection. All aggregated data relevant to the study are included in the article or uploaded as supplementary information.
